# ‘My wife is my doctor at home’: A qualitative study exploring the challenges of home-based palliative care in a resource-poor setting

**DOI:** 10.1177/0269216320951107

**Published:** 2020-09-18

**Authors:** Yakubu Salifu, Kathryn Almack, Glenys Caswell

**Affiliations:** 1 International Observatory on End of Life Care, Division of Health Research, Faculty of Health and Medicine, Lancaster University, Lancaster, Lancashire, UK; 2 Communities, Young People and Family Lives, Centre for Research in Public Health and Community Care, School of Health and Social Work, University of Hertfordshire, Hatfield, Hertfordshire, UK; 3 Nottingham Centre for the Advancement of Research into Supportive, Palliative and End of Life Care, Faculty of Medicine & Health Sciences, University of Nottingham, Nottingham, UK

**Keywords:** Family caregiver, Ghana, homecare, qualitative research, long-term care, palliative care, advanced prostate cancer

## Abstract

**Background:**

Family caregiving is common globally, but when a family member needs palliative and end-of-life care, this requires knowledge and expertise in dealing with symptoms, medication, and treatment side effects. Caring for a family member with advanced prostate cancer in the home presents practical and emotional challenges, especially in resource-poor contexts, where there are increasing palliative cases without adequate palliative care institutions.

**Aim:**

The study explored palliative and end-of-life care experiences of family caregivers and patients living at home in a resource-poor context in Ghana.

**Design:**

This is a qualitative study using thematic analysis of face-to-face interviews at two-time points.

**Participants:**

Men living with advanced prostate cancer (*n* = 23), family caregivers (*n* = 23), healthcare professionals (*n* = 12).

**Findings:**

Men with advanced prostate cancer face complex issues, including lack of access to professional care and a lack of resources for homecare. Family caregivers do not have easy access to professional support; they often have limited knowledge of disease progression. Patients have inadequate access to medication and other practical resources for homecare. Caregivers may be overburdened and perform the role of the patient’s ‘doctor’ at home-assessing patient’s symptoms, administering drugs, and providing hands-on care.

**Conclusion:**

Home-based care is promoted as an ideal and cost-effective model of care, particularly in Westernised palliative care models. However, in resource-poor contexts, there are significant challenges associated with the implementation of this model. This study revealed the scale of challenges family caregivers, who lack basic training on aspects of caring, face in providing home care unsupported by healthcare professionals.

**What is already known about the topic?**

- Home/family caregiving can be burdensome even when patients and their caregivers receive professional support.
- The challenges of palliative care are myriad, involving physical, practical, and emotional challenges.
- The main barriers to the provision of palliative care in developing countries include inadequate access to care both in the community and in the hospital, lack of coordination of care, and absence of insurance cover required to pay for healthcare.

**What this paper adds?**

- It provides an understanding of the challenges of care experienced by men living with prostate cancer and their family caregivers in a resource-poor context. Family caregivers become the ‘doctors’ of men with prostate cancer at home, assessing and managing care with little professional support.
- Family caregiving for patients needing palliative care is characterised by uncertainty about the progress of the disease and a process of ‘trial and error’ to establish what works in terms of care.
- Despite the challenges, caregivers see the provision of care at the end of life as reciprocal. Although managing symptoms can be associated with feelings of distress, the sense of filial obligation and reciprocity of care forms a basis for the creation of compassionate communities and home-based networks; an approach that needs consideration in palliative care in such setting.

**The implication for practice, theory or policy**

- Resource-poor settings would benefit from a multidisciplinary palliative care team to support care at home, provide information to patients and caregivers, and to give frequent feedback and help family caregivers in identifying and managing symptoms.
- The desire by families to provide care regardless of the challenges they face provides a sound basis for implementing a public health approach in palliative care through establishing compassionate communities that bring families/neighbourhood and community networks into the care of dying. Therefore, health care professionals could build on family caregivers’ willingness to participate in patient care, to coordinate and provide more effective and efficient care including the use of telemedicine.
- Access to resources to support this population needs to take into account of the health care literacy and poor internet access in resource-poor settings, so, Western solutions such as online resources or printed materials may not be adequate solutions.
- Efforts to integrate palliative care into mainstream healthcare and coordination with community health care staff to provide support to families appear essential.

## Introduction

Providing home-based palliative care and managing symptoms at home continues to be an issue in palliative care, even in developed countries where formal support exists.^1,2^ Specialist palliative care in home settings may be beneficial to patients and caregivers across different settings and in many countries.^3^ Even with professional support, home-based care can result in substantial emotional, social, and physical demands on informal caregivers.^4,5^ These demands increase in resource-poor contexts where there is little or no professional support for informal home-based care.^5^

There has been some substantial progress in palliative care services in Africa in recent times, notably in Uganda, Rwanda, and South Africa. However, palliative care on this continent remains underdeveloped and unevenly distributed due to lack of access to opioids, palliative care education, and fewer facilities that offer palliative services.^6,7^ A current comprehensive overview of palliative care in Africa shows isolated and limited provision of palliative care services in Ghana^8^ and empirical studies from neighbouring West African countries and emerging studies in Ghana, have indicated the critical functions of families in end of life care.^9–11^

Prostate cancer disproportionately affects black men,^12,13^ and advanced prostate cancer (stage III or V) does not only affect the individual with the disease but also has subsequent impacts on those living with them. The family plays a pivotal role when acting as key caregivers for family members with a debilitating illness.^14^ Previous research highlights the critical role of the family in the provision of care at home in the absence of palliative care professionals’ support.^15–18^ Additionally, there is an implicit cultural understanding that caregiving is considered the obligation of the family.^19^

Globally there is strong evidence of the challenges that family members face^20,21^ in providing care in situations even where they might have access to some professional support and advice. For example, family caregivers feel exhausted, and they may also be responsible for the care of their children,^22^ feel distressed,^23^ and feel they need support to carry out care at home.^24^ Grande and colleagues^21^ demonstrate that through their unpaid work, family caregivers contribute substantially to the economy of their respective countries and, in some cases, to the detriment of their own health. Family caregivers provide emotional support, intimate care, and direct hands-on care. They constitute what Abel et al.^25^ (p. 385) call the ‘inner network’ of the patients. However, in Abel’s model, there are further circles of care, which, at best are scarce and, at worst absent, in Ghana. These further circles include outer circle (extended family members, friends and neighbours), community network, service delivery and policy that together provide support for patients who need palliative care.

In resource-poor countries such as Ghana, there are additional challenges for family caregivers providing palliative care. There are not enough inpatient hospice/palliative care facilities so this is not an option for the majority of Ghanaians. However, there are also not enough trained staff to provide professional palliative care at home.^26–28^ A meta-ethnography of qualitative evidence highlights the importance of access to professional palliative care at home, which are found to reassure and support patients and family caregivers when facing life-limiting illnesses, by providing competent care, being present and able to advise on issues such as pain management, hydration and nutrition.^17^ This meta-ethnography included 19 studies from Sweden, the UK, USA, Australia and Denmark, which provided specialist or intermediate palliative care at home. This kind of support is not available in Ghana. It is rare for hospital palliative care teams to do home visits because they do not have enough staff and travel to remote areas is difficult due to the long distance from the hospital. Where visits do happen, staff pay their own travel expenses. Patients who do not have access to palliative care experts other than via occasional out-patient appointments must rely heavily on care provided by family members.^29,30^

## Aim

To explore the understandings and experiences of men living with advanced prostate cancer and their family caregivers about the challenges of being cared for and providing informal care in the home in a resource-poor context, where palliative care services are under-developed.

## Method

Qualitative repeat (6–12 weeks interval) interview study with patients and family caregivers, and focus groups with health care professionals. Social constructivist theory and interpretivism underpin this study where co-creation of knowledge and ideas are the product of the interaction between researcher and participants.^31,32^ To ensure a standard of reporting qualitative research findings, we followed the consolidated criteria for reporting qualitative studies (COREQ) guidelines.^33^

### Setting

The setting was across Ghana, and recruitment took place in a tertiary hospital that has one of the only two functional facility-based palliative care teams, where patients attended out-patient appointments. This site enabled the use of research nurses to identify and approach men with advanced prostate cancer who met the inclusion criteria for the study.

### Participants

Twenty-three men with advanced (stage III or V) prostate cancer, 23 family caregivers, and 12 health professionals participated; this paper reports on the perspectives of patients and family caregivers.

### Sampling

**Figure 1.**
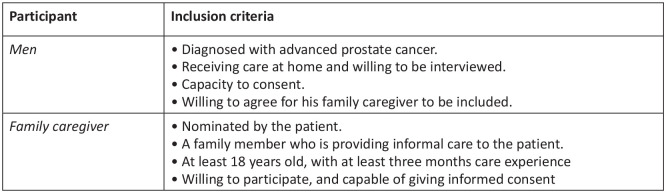
Eligibility criteria of participants.

Purposive sampling was used to recruit participants who met the inclusion criteria (see Figure 1) until a sufficient number that comprehensively explored the participants’ experiences.^34^

### Recruitment

Staff at the Oncology Department gave potential participants information about the study during out-patient appointments. For those who did not have sufficient language skills, the staff explained the information sheet in the native language. Participants had two weeks to think about it and to report their decision to the team. Interested participants were then referred to the researcher, who further confirmed their consent. The men then nominated their family caregivers to participate in the study.

### Data collection

YS undertook all the fieldwork in Ghana from November 2016 to May 2017. The men and their family caregivers took part in up to two repeat interviews at approximately two-month intervals, facilitating rapport between researcher and participants and enabling discussion of any changing experience.^33,35^ The interviews were semi-structured around what their experiences were and how they managed care at home, allowing flexibility to explore issues raised by participants while ensuring core topics were covered.^36–39^ The interviews took place mainly at home through participants’ choice and focusing on participants either as individuals or pairs;^40^ details in Table 1. The topic guide for the patients’ and caregivers’ interview is presented in online supplemental material.

**Table 1.** Data collection methods.

Method	First phase	Second* phase	Total interviews
1. Individual Interviews			
a) Patients	12	9	21
b) Family caregivers	12	9	21
2. Dyad/joint Interviews	11	10	21
**Total interviews**	**35**	**28**	**63**

* Due to the death of 4 men, only 19 men and their families took part in the second interview.

Most of the interviews were conducted in the local Ghanaian language. Although the first author is bilingual, linguistic experts verified the translated transcripts to ensure accuracy.^41^ For instance, the verification provided a perfect translation of some words and phrases from the Ghanaian language into English.^42,43^ All the interviews were preceded by the completion of demographic information. With participants’ permission, conversations were audio-recorded and transcribed. Written field notes, made once the researcher had left the field, were taken into account during analysis for contextual details.

### Analysis

Qualitative Data Analysis Software, NVivo 12 ©, was used to manage the data. We used thematic analysis to identify, analyse, and report common themes.^44^ The first author did line-by-line coding of all transcripts, reading, and re-reading them to ensure all the salient points were captured. Codes were derived from the data rather than using a previously conceived coding framework in order not to truncate emerging ideas. Then during discussions among all the three authors, the coding framework was reviewed, discussed as it was applied to the dataset.^45^ Pseudonyms have been used in the excerpts to protect the identity of the participants.

### Ethical considerations and informed consent

The University of Nottingham’s Faculty of Medicine and Health Sciences research ethics committee approved this study (reference no. F12092016). The study was registered at the hospital where recruitment took place, and a certificate of registration was obtained (Registration number: RD/CR16/247). Ethics committee approval was also received from the Committee on Human Research, Publications, and Ethics at Kwame Nkrumah University of Science and Technology (Ref no. CHRPE/AP/496/16). Interviewing participants about such a sensitive issue was sometimes an emotional experience.^46^ Informed consent was obtained from all participants. There was no relationship between researchers and the participants, and only YS had access to identifiable information.

## Findings

Forty-six participants took part in the study’s first phase, and 28 participants were followed-up during the second round of interviews, producing 63 interviews in all, with 21 joint interviews. The interviews lasted 1 h on average (45 min to 2 h). The breakdown of all participants is as follows: men living with prostate cancer (*n* = 23) and family caregivers (*n* = 23), as shown in Table 2.

**Table 2.** Demographics of the participants.

Men living with prostate cancer	
*Age (years)*	# Participants (*N* = 23)
49–58	6
59–68	10
69–78	4
79–88	3
Total	23
***Type of palliative treatment*** ^1^ ***at the hospital***	
Hormone treatment	15
Surgical Castration (2)/ Radical prostatectomy (4)	6
Radiation	4
No longer being actively treated	4
***Level of education***	
Nil	8
Primary	5
Secondary	2
Tertiary	8
***Occupation***	
Nil	3
Teacher	1
Farmers	3
Retired (both mandatory & voluntary)	8
Traders	4
Priest	1
Artisans	3
Family caregivers	(N =23)
***Relationship to patient***	
Wife/spouse	10
Child/Children	9
Mother	2
Sibling	2
***Years of experience working as a caregiver***	
<*1 (less than 1)*	12
1–2	7
3–4	3
5^+^	1

1. some of the men received more than one treatment.

Three main themes from the data are the practical and emotional issues about care at home, caring in the absence of the support of health staff, and pain management, as detailed in Table 3. The principal findings are presented in Table 4.

**Table 3.** Family caregiving and challenges of accessing care and provision of home-based family caregiving.

*Practical and emotional issues* • Managing sudden change in condition and coordinating care • Conflict in care provision • Significance of providing food for patients *Navigating care at home* • Getting on with care (trial and error) • Reciprocity: A duty to fulfil *Pain management* • Assessing pain • Access to pain medications • Use of herbal medicines

**Table 4.** Key findings.

*Key Findings* • In a resource-poor setting, professional support for family caregivers and patients to manage care at home is lacking. • Family caregivers face enormous practical and emotional challenges, such as managing conflict in care provision, a sudden change in the patient’s condition, or dealing with the refusal of food. Patients and caregivers have difficulty accessing the services they need. • Family caregivers learn how to provide care by trial and error, and this affects the quality of care they provide, as well as the quality of life of the men living with advanced prostate cancer. • The cultural belief in the reciprocity of care motivates families to maintain caring even if they are struggling to cope. • Family caregivers assess and manage pain and medication side effects at home unsupported. • The use of herbal medications in place of or in conjunction with other medicines from the hospital is common, and this could have a detrimental effect on the patients, perhaps than the prostate cancer symptoms.

## Family caregiving and challenges of the provision of and access to supportive care

Several challenges relate to the difficulties of accessing professional palliative care services and the need for informal home-based family caregiving for men with advanced prostate cancer. These challenges included the practical and emotional issues of care, how care is carried out at home, and pain management. The findings also highlight cherished cultural values, such as belief in the reciprocity of care and filial obligations to care that is important for the growing field of global palliative care.

### Practical and emotional issues

#### Managing sudden change in condition and coordinating care

A key concern highlighted by a majority of participants was deep feelings of anxiety and fear related to the unknown outcome in terms of deterioration towards the end of life and death, and how this affected the general wellbeing and life of the patient and family caregivers:

*His condition became worse. I didn’t know what was going on. I’ve been managing him at home, but what happened about two weeks ago, I was frightened to death [he collapsed, and his eyes were staring at the sky]. (Eno, Mike’s wife)*

Unexpected changes in patients’ conditions were sometimes misunderstood to mean that the patient was near his end of life. Caregivers also interpreted changes, such as breathlessness and a period when the patient was less active, as them having almost died. Caregivers did not know what to do in the absence of advice from a healthcare professional:

*He ‘dies’ and ‘resurrects’ most of the time. His condition has thrown us [the family] into a state of confusion. We don’t know what to do. (Sophia, Samson’s caregiver)*

*It’s terrifying. I feel like dying. I am. I don’t know how my condition is going to end. (Mike, patient)*

Families have to cope with sudden changes in the patient’s health without adequate knowledge of what to expect and what to do; it is not surprising therefore that caregivers (and patients) encountered frightening scenarios bound up with the emotions of the patient being a member of their family.

Caregivers identified a wide range of tasks they undertook in coordinating care. Such coordination mainly involved, but was not limited to, arranging transport to go to the hospital in times of emergency or for follow-up appointments, and making hard decisions such as whether to spend scarce resources on food or medicine. The organisation of care tasks often had to fit around family caregivers’ other roles or work commitments:

*. . .it’s not comfortable doing that all by myself. I need to ensure that I direct the affairs relating to care to avoid confusion and all that [*addressing different duties all at the same time]. *The task is not as simple as that. (Samuel, Tawiah’s* son)

#### Conflict in care provision

Sometimes there was a conflict between family members about how care should be provided. For example, whether to use orthodox or alternative traditional medicine could be a topic of contention. Where such conflicts were unresolved, it had a negative impact on caregiving:

*Some were pushing him to undergo chemotherapy while others vehemently opposed to it. Therefore, we are divided about what to do, and the other faction (who believe they use herbal medicines) are not happy and not helping with his care. (Kwaku’s wife, Okonore)*

In some cases, there were conflicts between what the patients wanted, what caregivers could provide, or what they felt was suitable for the patient, and this affected the quality of care provided and experienced. Some patients refused food as a protest for unresolved conflict regarding their care.

The challenge of patients’ refusal of food and the impact on family life were raised. General symptoms of end-stage cancer as well as some of the side effects of chemotherapy reported in this study (*n* = 14) include nausea, vomiting, and loss of appetite. This meant some patient participants refused to eat or only ate small amounts of food. Caregivers expressed apprehension about the refusal of food:

*Our main problem is that he doesn’t eat enough. He needs to eat to get active and for us, too, to get the appetite to eat. If he refuses food for days, everyone is bothered too. (Eno, Mike’s wife)*

#### Significance of providing food for patients

Refusing food or eating just a little was a challenging everyday experience for families managing the care of men with advanced prostate cancer. In Ghana, food has cultural significance; when a husband refuses to eat the food his wife prepares, it is interpreted as the husband having a dispute with his wife. Therefore, such arguments between husband and wife are regarded as inappropriate and go against cultural expectations of harmony in a marriage. However, other relatives also found the refusal to eat difficult to understand:

*I am unsure if I have pissed him off or something. I don’t figure out why he refuses his best food. I can’t tell. He might be annoyed, or he is doing that intentionally to end his life. (Sabi, Boat’s brother)*

Sabi’s sentiment was another dimension of the difficulty of dealing with the refusal of food by his brother, Boat. Sabi’s narrative was an emotional response to this refusal of food, as Sabi was trying to work out whether his brother was starving himself deliberately or refusing food to demonstrate annoyance with his brother.

### Navigating care at home

#### Getting on with care (trial and error)

Lack of formal services to support the provision of care at home was a key challenge that participants identified. Some were explicit that they did not feel competent to provide the necessary care – identifying, for example, not having the requisite practical skills. Many reported using a process of ‘trial and error’ to develop their caring skills.

*It’s mostly trying one thing or the other to see which one works best. We do ‘trial and error’ most times honestly. We are on our own when we are at home. Healthcare is not my field of training; mine is in accounting, and I don’t know how to nurse big wounds. (Teiko, Norbert’s son)*

Any problems which arose when caring for the patient at home fell on family caregivers for resolution. They usually had no experience or relevant training, and the family caregiver, therefore, served as a physician for the patient at home:

*I must be thankful to the doctors for their excellent work at the hospital and for helping us to live. I can’t thank my wife enough. My wife is my ‘doctor’ at home, ensuring that I get all the care I need. (Baabamu, patient)*

The ‘how to care’ dilemma included practical care, how to minimise pain and increase comfort, how to prevent complications, among other things. For instance, Efo’s caregiver reported:

*He is very heavy for only one person to provide personal care. He has a big sore in the lower back. . . . . . It’s difficult for us (caregivers) to avoid this if we can’t anticipate and know what to do. (Mawuli, Efo’s grandson)*

In the absence of palliative care professionals managing and coordinating the care at home, family caregivers planned and executed care while not necessarily feeling competent to do so. Feeling isolated without recourse to professional advice in the difficult situation of dealing with the advanced life-limiting disease at home was, in itself, a frightening situation for family caregivers. The ‘how to care’ was a challenge for some patients as well:

*. . . . . the doctor me told I had infections in my penis and my sacrum. We don’t know how to prevent or treat this at home. (Babaamu, patient)*

Some family caregivers tried to seek hospital admission when the patient’s needs and symptoms felt too challenging to manage:

*Anytime my husband is discharged from the hospital, we take charge at home doing everything; because no health staff has ever come home to assist or something. If my husband’s condition becomes poorer, we then send him [back] to the hospital for a few days for management. (Abiba, Maegyida’s wife)*

Abiba, in the quote above, highlighted the only option open to her to address difficult situations she was not capable of handling at home. This family had their private transport to facilitate them getting Maegyida to the hospital when the need arose. However, not all families had access to sufficient resources to enable this to be an option.

#### Reciprocity: a duty to fulfil

There was frequent mention of the fact that family caregivers have a duty to fulfil in providing care for the patient as a family member. Reciprocity was understood from several points of view. First, there was the general belief that the ‘father’ is the spiritual head of the family and that there is an obligation to ensure that he is well cared for. Second, caregivers saw caring as a time to give back the love and sacrifices which their relative has made and continued to make in their lives. Third, there was also the belief that caregivers were providing the care to these men so that if they fall sick themselves in the future, their children or family members will not leave them to their fate. The fourth reason for reciprocity was the fact that Ghanaian society considers it inappropriate to neglect the sick, so the family is obliged to provide care. Finally, there is a cultural belief of punishment in the next life if a family neglects the sick. Several family caregiver participants referred to notions of reciprocity:

*We want to show him love and support by giving back what he did for us when we were young and fragile. (Agyei, Gyasi’s son)*

*We are also men; a similar thing (prostate cancer) might happen to us. You can’t tell what will happen. Can you? (Karikari, Atta’s son)*

In one instance, a patient’s son did not refer to reciprocity as his key motivation but rather resented his father’s past behaviour:

*He is my birth father. He refused to send me to school even though he had the means to do so. I have decided to teach him how it feels like when someone rescues you timely. If I think about those past events, I wouldn’t have been here to care for him. (Joobu, Asamoah’s son)*

Here, Joobu implicitly claimed the moral high ground, treating his father as he believed his father should have treated him in the past.

Caregiving could be interpreted as giving back some of the love family members had received in the past. Providing care could also mean avoiding societal disapproval for deserting the sick:

*I must be with him, especially this time he needs me the most, for better or worse, and in sickness and life. If I abandon him, I know I have offended the God I serve. (Achiaa, Opanin’s wife)*

*I must care for him. God will not forgive me if we abandon him because he (dad) has done a lot for the family and me. I can’t even stand the criticisms from others. Everyone knows what my dad has done for us growing up. (Sophia, Samson’s daughter)*

Reciprocity was underpinned by love, religious or cultural beliefs, and giving back or anticipation of getting similar support.

### Managing pain at home

#### Assessing pain

Participants reported the challenges of pain management. Assessing pain was a joint decision by patients and their caregivers. For most patients, the pain was sometimes unbearable and relatively constant:

*It’s very severe pain, and I scream like a woman in labour (Boat).*

*Hmm, the pain is very unbearable sometimes. The last time I cried. That made my wife and children also followed suit as if we were mourning (Nobert).*

As there was no tool to guide pain assessment, caregivers were unable to assess when to give or repeat a pain medication when the patient complained of pain. One caregiver expressed her frustration about dealing with pain at home:

*You know, it’s hard. After giving all the medicines to him, and he still complains of pain. When it happens like that, I am entirely at a loss (Ivy, Nii’s daughter).*

In some instances, the men suggested that some complaints about pain were ignored or minimised:

*After I went for the procedure, when I came home that night, I couldn’t sleep at all. I was awake all night. I rested a while in the morning, and the pain came again. I complained. I asked for stronger pain relief, but it was ignored. Yes, it was. They (caregivers) think I might be demanding unduly (Nelson, patient).*

#### Access to pain medications

For most participants, access to pain medication and dealing with medication side effects were challenging. They reported a regular shortage of pain medications such as morphine at the hospital to take home for use:

*I prefer to get the medicines at the hospital where I go for review (tertiary hospital) because I am guaranteed of its effectiveness. But sometimes, they too, they run out of stock. (Ali, patient)*

Acquiring morphine outside the hospital was problematic, and sometimes participants reported having to buy sub-standard medications that they found did not effectively control pain. Consequently, patients and their family caregivers felt helpless about unrelieved pain. Some caregivers expressed distress, resulting from confusion as to what to do at home when a patient’s condition worsens.

*It has been a vigil night for us for the past few days, and we all were panicking. I didn’t know what to do after he complains even after giving him all his pain medicines [Tears flowing]. (Safia, Boat’s caregiver)*

#### Use of herbal medicines

Some patients made use of herbal medicines. There were a number of factors to account for this, including a lack of supervision from health care professionals plus poor access to and provision of palliative care. Some patients and caregivers thus resorted to traditional herbal medicine, which is readily available within the communities. Some participants believed that herbal medicines might be more effective in managing their symptoms:

*We got some herbal medicines from a woman who is known to be an expert in that field. (Eno, Mike’s wife).*

*I went to (name withheld), and I got those medicines because they said when I use them, I wouldn’t need to have the catheter in place. I paid a lot. After some time, my problem became worse (had sores at the anus). I went there again, and still, my problem wasn’t solved. I had to stop and report to the hospital (Ofori, patient)*

Some diseases or symptoms were regarded by participants as ‘unnatural’ and ‘not treatable by doctors’; hence, they felt the need to seek alternatives that might be more effective:

*I took some herbal medicines (forgotten the name) to treat cancer. I stopped when I have sores in my anus (Opoku, patient).*

Other men used herbal medicines alongside conventional treatment from the hospital to treat sexual weaknesses:

*I used some herbal medicines to treat my erectile problem. It was ok at first, but now the problem is worse (Maegyida, patient).*

Although some herbal medicines are duly registered and certified for the treatment of some diseases, patients and caregivers resorted to alternative medicines that were easily accessible, but the efficacy and health effects could not be ascertained.

## Discussion

This study highlights the lack of access to formal/professional palliative care and the consequent reliance on families at home in Ghana. The findings have relevance to Ghana and other resource-poor countries where palliative care is under-developed.^6,7^

Even where palliative care services are well-established, and there is recognition of the need to support caregivers, caring for a family member at the end of life can be emotional and challenging in practical terms.^1,15,20^ This study provides insights into the additional challenges of home-based family care for men living with advanced prostate cancer in the resource-limited context. The findings highlight that home care for advanced prostate cancer is challenging, and multi-faceted. It is associated with stress and anxiety when caregivers do not know what to do at home, unsupported by professional staff. Thus, the lack of resources for coordination and support from healthcare professionals creates a gap in the provision of accessible, effective, and holistic care.

Providing meals can be an expression of care and love.^47^ In our study, caregivers found it difficult when the patient refused food. Additionally, there is no access to alternative ways to ensure the patient gets adequate nutrition at home. There is a small but increasing body of research in the developed world that addresses the financial impact of family caregiving in a palliative care context.^48,49^ Families in Ghana have additional costs as they have to absorb the cost of medicines and other equipment needed to care for the patients at home.

The findings highlight that most caregivers have no prior experience of caring for someone with palliative care needs. This reflects other research that suggests that caregivers can, over time, become knowledgeable and efficient through their experiences of caregiving or by observing how others do care.^17,50,51^ Caregiving is a shared responsibility for the family.^52^ The family creates a place where men feel safe and supported despite the challenges of home-based care.^1^

A sense of ‘giving back’ to a family member, especially members who are regarded as heads of family units, provided a key motivation for providing care. As discussed, family caregivers also reported an underpinning moral duty to provide care due to the fear of living with guilt if the patient died due to their neglect^11,53^ or the patient committing suicide due to self-stigma.^53^ Reciprocity of care and what this means to the caregivers’ sense of giving back to their loved ones are highlighted in other studies,^10,51,54^ and this is underpinned by a strong cultural/religious belief^55^ as is the idea that family caregivers must be supported.^56^

Pain management is a dominant feature of palliative care,^57^ and this is particularly complex in resource-poor settings where access to morphine is problematic.^58^ The main pain medications used for treating moderate to severe pain in Ghana are morphine and pethidine, which are classified as controlled drugs, which one cannot buy over the counter even with a prescription. Usually, pethidine is administered under the supervision of a trained practitioner at the hospital in cases of severe pain.

Three main problems that serve as a barrier to pain relief in this context are: first, patients have to pay for pain medications, and therefore, there is a financial barrier to access pain control medications. Second, the unavailability of pain medication^59^ (some hospitals run out of stock due to non-payment of health insurance claims), and the patients have to buy from private pharmacies. The third issue is that caregivers have to administer pain relief and are not confident about doing so or how much to give. This finding is consistent with other studies, which indicate that pain is complex and dynamic to assess, especially at home unsupported by health care professionals, where access to and effectiveness of pain medications are challenging^57,60^ and some patients cannot afford the cost of pain medications.^61^

This study noted that many participants used alternative health treatments such as herbal medicines to manage their condition, or they were used alongside orthodox medicine. Patients have to pay for medications, and sometimes this means they do not have adequate access to the medication and other care supplies needed at home. Patients reported the use of traditional herbal medicine, often advertised on buses, marketplaces, church premises, and mosques. Sellers claim the medicines can enhance sexual performance, cure erectile dysfunction, treat pain, or treat cancer ‘completely’.^62^ Many patients, therefore, stop visiting the hospital or use these traditional herbal medicines in addition to any medication prescribed by doctors. There are dangers that without regulation or proper advice on use, herbal medicines might be detrimental to, or worsen their condition.^63,64^ Participants rationalised the use of alternative medicines, reporting that those medicines are cheaper to buy and more easily accessible than the prescribed medications. However, as noted, the efficacy of such alternatives is not known in all cases, and participants often reported adverse responses or reactions to traditional medicines they had used. This highlights the need for such medicines to be strictly regulated, supervised, and for health care professionals to encourage open discussions with patients and caregivers on their use of such medicines.^65^

### Strengths and limitations

The strength of this study is that it is, to our knowledge, the first study exploring informal home-based palliative care in Ghana from the perspectives of patients, family caregivers, and healthcare professionals. The use of serial interview design ensured an in-depth and holistic understanding of the experience of home-based care provided by family members.^66^

Coding was done by the first author (YS), a native of Ghana. This may have introduced some bias, which is a potential limitation. As noted, we attempt to ameliorate any potential bias by rigorous discussions of the findings among the three researchers, two of whom (KA and GC) have no background culture from the country. Any taken-for-granted or preconceived notions about informal home care in Ghana were thus picked up and discussed. The decision to recruit participants from only one of the two palliative care centres was made based on the practicality of the research due to the large size of the country, but this may be a limitation of this study. However, we believe that issues raised by participants may be similar to those in the southern half of the country.

### Implications for policy and practice

In the absence of professional palliative home services, a support network for informal caregivers who are providing hands-on care for men living with advanced prostate cancer at home (and for all caregivers providing care for family members at the end of life) would be invaluable. Such a support system could be strengthened by developing this in partnership with healthcare professionals to achieve the new public health approach to palliative care, compassionate communities.^29,67^ Further steps are needed to improve palliative care, primarily home-based family caregiving, in resource-poor countries.

*First, promoting palliative out-reach projects.* Efforts to empower healthcare professionals to gain/consolidate their palliative care experience through means such as *care navigators* would help provide timely supportive care. With this, health care professionals could be assigned with catchment areas to regularly visit to discuss patients’ conditions and support them with care.

*Second, developing social and health care policy, including* a guiding framework to ensure the delivery of coordinated and quality home-based care to men with advanced prostate cancer.^68,69^ This guidance could draw on the informal support systems and belief in the reciprocity of care, taking a bottom-up approach to care.

Telemedicine, a concept of providing virtual care via online, is operating in other countries with vast geographical areas that can be difficult to navigate, and where access to health care is an issue such as parts of Australia, as well as other African countries.^70–72^ It is also used elsewhere, especially for delivery of out-of-hours telephone support and video conferencing for interactive case discussions.^73^ The online resources for caregiving are dependent on internet access; therefore, in places where some communities have poor access to the internet, a telemedicine technology which provides both online and offline resources for family caregivers could be helpful.^74^

#### Implications for future research

This study has identified significant challenges in providing informal home palliative care unsupported by health staff. The World Health Assembly passed Resolution WHA67.19 in 2014, urging nations to take a palliative care approach, mainly community/home-based care, to all their citizens by integrating its principles into all aspects of health and social care.^75^ There are assumptions that one palliative care model fits all, but this research shows that is problematic. It is, therefore, essential for further research to explore the role of compassionate communities in supporting care at home, and the feasibility of telemedicine that could be useful in such settings even during a pandemic crisis such as COVID-19.^76^ In the longer-term, research could assess the feasibility of using telemedicine and other forms of palliative outreach services.

## Conclusion

Palliative care services are limited, and in the early stages of development in the study’s context. Given the geography and poor infrastructure, such as poor internet access, the answer does not lie in a straightforward application of Westernised palliative care models, but we argue that family caregivers need to be better supported. In the meantime, satellite palliative care services, the use of telemedicine, or the sharing of information on mobile apps could support patients and their family caregivers to navigate care at home. For any potential online resources, this should take into accounts the poor internet access and literacy rate.

There is a clear need for person-centered care that is coordinated by both professionals and family caregivers. This could be supported by a comprehensive health and social care policy underpinned by significant resources to facilitate the development of home-based care delivered by health care professionals in the long term. Training of healthcare professionals to provide appropriate support for this population while relying on the existing family support networks is required. We call for the building of compassionate communities and home-based networks for the care of the dying, such as those being advocated in public health approaches to palliative care that is tailored to the context.

## Supplemental Material

6._Topic_guide_ – Supplemental material for ‘My wife is my doctor at home’: A qualitative study exploring the challenges of home-based palliative care in a resource-poor setting Click here for additional data file. Supplemental material, 6._Topic_guide_ for ‘My wife is my doctor at home’: A qualitative study exploring the challenges of home-based palliative care in a resource-poor setting by Yakubu Salifu, Kathryn Almack and Glenys Caswell in Palliative Medicine
