# Supplementary material for: ‘My wife is my doctor at home’: A qualitative study exploring the challenges of home-based palliative care in a resource-poor setting
Source: Palliat Med. 2020 Sep 18;35(1):97–108. doi: 10.1177/0269216320951107 (PMC7797613; doi:10.1177/0269216320951107)
Supplement: 6._Topic_guide_ – Supplemental material for ‘My wife is my doctor at home’: A qualitative study exploring the challenges of home-based palliative care in a resource-poor setting [file 6._Topic_guide_.pdf]

A Topic guide

**a) Interview guide-patients with prostate cancer**

Can you please walk me through your experience after the cancer diagnosis and living with this condition?

How do you manage the condition at home, and who are those involved in most of the care?

Could you please tell me how the care is done? What role do you play personally?

How do you get information about your condition and how it is managed?

How satisfied are you with the care at home?

Please tell me more about the resources and support that are available for the care. (Probe: Emotional, practical, financial, etc.)

Can you please share common challenges or barriers that are affecting the care at home?

How can care be improved in your own opinion?

**b) Interview guide-Family caregivers**

Please tell me about yourself as the caregiver and how you became a caregiver

Can you walk me through how your day is like regarding the care you provide? Probe

How do you provide the care, and how will you describe such experience?

(Probe: logistics for care, how and where they get it, etc.)

How do you navigate your way through the care and your personal life? Tell me more

Tell me a little more about some experience that helps with the care

How do others contribute to the care (family members, HCP, religious bodies, government, NGO, etc.), and what exactly do they do?

In every activity, there might be challenges one way or the other. How have you confronted some challenges regarding care?

Probe: How is the conflict, if any, arising from the care managed (between caregiver, patients, HCP, and other family members?)

Are there any challenges (Probe: how to care, informational need, support, financial, professional, emotional, etc.)

Are there some approaches you think could be done to improve care? And what is expected)
